# Marsupialisation of 12 odontogenic cysts in Boxer dogs: Retrospective case series

**DOI:** 10.3389/fvets.2023.1099128

**Published:** 2023-01-17

**Authors:** James Haseler, Ingrid Tundo, Pete Southerden

**Affiliations:** Part of Linnaeus Veterinary Limited, Department of Dentistry and Oral Surgery, Eastcott Referrals, Swindon, United Kingdom

**Keywords:** marsupialisation, odontogenic cysts, Boxer, cysts, retrospective study, computed tomography, volume measurements, case series

## Abstract

Marsupialisation of odontogenic cysts is a minimally invasive treatment method used in human dentistry. Marsupialisation decompresses the cyst and promotes remodeling of alveolar bone and shrinkage of the cyst. In this retrospective study we look at the effectiveness of marsupialisation at reducing the size of odontogenic cysts in dogs. The case series consists of six Boxer dogs with 12 odontogenic cysts. Each case underwent a high resolution CT scan prior to treatment and at follow-up. Each CT scan was reviewed, the volume of each cyst calculated using manual segmentation and the reduction in cyst volume calculated. There was a marked reduction in cystic volume of 66.6% over a mean of 138 days. This shows that the use of marsupialisation effective method of reducing cyst volume.

## Introduction

An odontogenic cyst is a pathological, epithelium lined cavity which is derived from the odontogenic epithelium and contains either fluid or semisolid material (1, 2). Odontogenic cysts occur in tooth bearing regions of the jaw (2) and over time can create a large bony defect as a result of fluid accumulation and pressure necrosis of the surrounding bone (3). The expansile nature of odontogenic cysts causes weakening of the surrounding bone and can cause complications such as pathological jaw fracture if the cyst is located in the mandible. Cysts within the maxilla can expand into the maxillary recess or nasal cavity (3, 4).

In veterinary dentistry, the current treatment method of choice for odontogenic cysts is the surgical exposure of the cyst and complete extirpation of the cyst lining through the use of curettage (5). This technique has been proven to be an effective treatment method for odontogenic cysts with little or no chance of recurrence (1, 6, 7). However, this method is invasive and in larger cysts, may require creation of a large surgical incision, extraction of multiple teeth and placement of a bone graft.

Marsupialisation was first described in human dentistry by Partsch (8). The process of marsupialisation involves creating an incision in the gingiva or mucosa overlying the cyst, raising a mucoperiosteal flap with the use of releasing incisions to expose the cyst. A section of cyst lining is removed, decompressing the cyst and the edge of the remaining cyst lining is sutured to the gingiva or mucosa, creating a stoma (9). The stoma allows drainage of fluid from within the cyst which facilitates regrowth of surrounding bone and reduction in the size of the bony deficit created by the cyst (4, 8). This method is useful for large cysts or in cases where extirpation of the cyst may damage vital structures (6). It is also useful when the cyst is multilocular and there may be a higher risk of incomplete extirpation.

To the authors' knowledge, there has only been one report in the veterinary literature of the use of marsupialisation in the treatment of an odontogenic cyst. In that study, this technique was used alongside extirpation in the treatment of an extensive periapical cyst in order to avoid excessive iatrogenic trauma to the maxilla of the patient (10). There is no documentation of this technique being used in a staged procedure for the treatment of large odontogenic cyst in dogs.

The objective of this retrospective study was to review a case series of Boxer dogs which had odontogenic cysts marsupialised as a first stage treatment and to evaluate the effectiveness of the technique at reducing the cystic volume. Volume reduction was calculated by comparing cyst volumes on computed tomography before marsupialisation and at follow-up.

We hypothesized that marsupialisation is an effective first stage treatment of odontogenic cysts and results in significant reduction in volume of the cyst through decompression, allowing safe enucleation at a later stage if required.

## Materials and methods

### Case inclusion

Clinical records were searched for Boxer dogs that had undergone treatment of odontogenic cysts at Eastcott Referrals, Swindon, UK, part of Linnaeus Veterinary Limited between 2019 and 2022. To meet the inclusion criteria, a case must have had at least one odontogenic cyst originating from the mandible or maxilla treated with the use of marsupialisation. Each case was required to have had a preoperative and follow-up high resolution CT scan as well as a histopathological diagnosis. The clinical notes for cases that met these criteria were reviewed, and the following were recorded: the signalment (age, sex, neutering status), presenting clinical signs, findings of clinical examination at presentation and follow-up, histopathological diagnosis and the interval between marsupialisation and follow-up CT.

### The marsupialisation technique

Since 2019 a standard protocol has been used at Eastcott Referrals, Swindon for the management of odontogenic cysts using marsupialisation. This protocol is described below.

A transverse collimated CT scan was obtained for each case prior to surgery using the following acquisition parameters (120 kVp, automated mA, 0.625 mm slice thickness, spiral pitch factor 0.53, 512 × 512 matrix, and smallest possible scan field of view). A bone and soft tissue window is acquired which allows us to locate the cyst, identify any teeth involved and a point of access to the cyst. Following this, the patient was positioned in lateral recumbency, and a detailed oral examination and dental charting was performed. The oral cavity was rinsed with a 0.12% chlorhexidine solution and appropriate locoregional anesthesia was performed.

**Figure 1:**
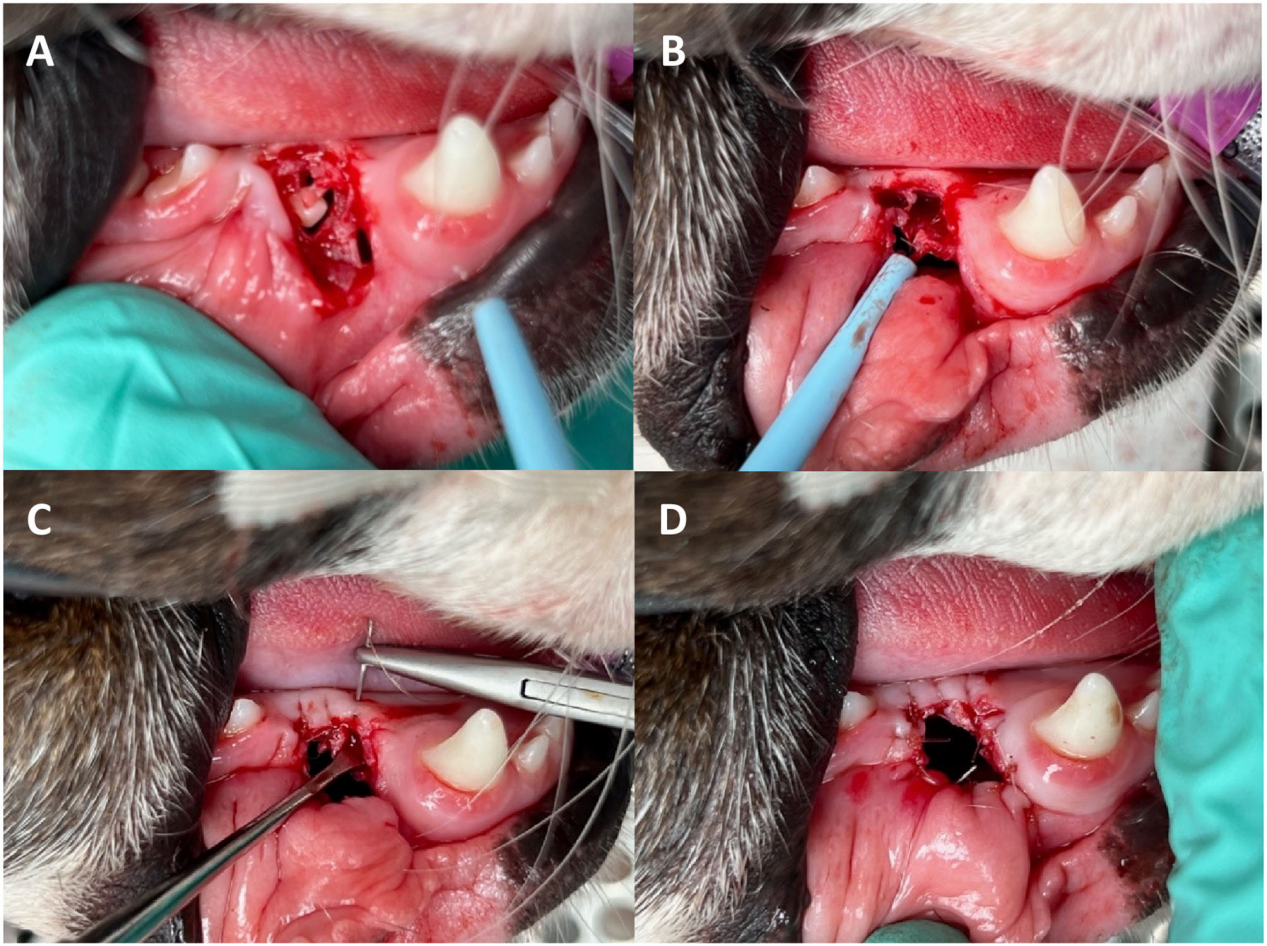
Marsupialisation of a dentigerous cyst at the level of an unerupted right mandibular first premolar tooth. **(A)** Creation of a triangle mucoperiosteal flap to expose the unerupted mandibular 1st premolar tooth and the cyst lining. **(B)** Unerupted tooth was extracted and the cyst lining separated from the alveolar bone. **(C)** Suturing the gingiva to the cyst lining. **(D)** Final marsupialisation.

Once the position of the cyst had been identified, the location and size of the marsupialisation was determined. The location of the marsupialisation was away from any important structures and where the cyst had minimal overlying soft tissue or bone. If the cyst was superficial and had areas only covered by soft tissue, the marsupialisation was created by incision directly into the cyst with a Swann Morton No. 64 Beaver blade. If the cyst was surrounded by bone, a large triangle or pedicle mucoperiosteal flap was created and elevated with the use of releasing incisions and an alveoloplasty performed to expose the cyst lining prior to incision. A large circular incision was made in the cyst lining and the tissue removed. This tissue sample was placed in 10% buffered formal saline and sent for histopathological analysis. The content of the cyst was removed with suction and the remaining lumen lavaged with copious amounts of 0.9% saline. In the case of a dentigerous cyst, the causative tooth was identified and extracted. If the teeth had excellent periodontal health, no evidence of external inflammatory resorption and at least the apical 1/3 of the tooth was encased in bone, they were left in place. If this criteria were not met, the teeth were surgically extracted. The edge of the remaining cyst lining was sutured to the surrounding oral mucosa and/or gingiva using simple interrupted 4–0 monofilament suture material (poliglecaprone 25/Monocryl^®^, Ethicon, Somerville, NJ, USA) creating a marsupialisation (Figure 1). The size of the initial flap and/or stoma was determined by the surrounding structures. A large stoma is preferred to help with visualization and limit the risk of premature closure during the treatment duration.

Each case was discharged with a 7 day course of meloxicam 0.1 mg/kg SID (Metacam™, Boehringer Ingelheim, UK) and acetaminophen/codeine (Pardale-V™, Dechra Pharmaceuticals, UK) 10 mg/kg TID. The owners were instructed to feed soft food only and lavage the stoma sites with 0.12% chlorhexidine solution twice daily after meals for 2 weeks. Hard chews and toys were also restricted for this period. Each owner was advised to book a follow-up appointment with the referring vet 10–14 days post-operatively. Following this, regular monthly check-ups were advised to monitor the stoma sites for signs of premature closure.

Each case is re-examined at the clinic 3 months post-operatively and a follow-up CT scan is performed using the same acquisition parameters. A detailed oral examination including dental charting is performed and the patency of the stoma is assessed using a periodontal probe. Appropriate local anesthesia is performed and the remaining cyst lining extirpated. In some cases where there is sufficient infilling of bone so that no cavity remains, the stoma was left open and the patient was re-examined in a further 3 months.

### Calculation of the cyst volume

Each CT scan was reviewed in DICOM format by both a board-certified radiologist and a resident in dentistry and oromaxillofacial surgery using viewing software (Horus, OsiriX^TM^, Geneva, Switzerland). For each cystic structure identified, the location was recorded as either mandibular or maxillary. The cyst was then categorized based on the surrounding tissue into either a cyst completely surrounded by bone (B), a cyst surrounded by bone with soft tissue involvement (B + ST), or a cyst surrounded by bone with extension into the nasal cavity (B + NC).

**Figure 2:**
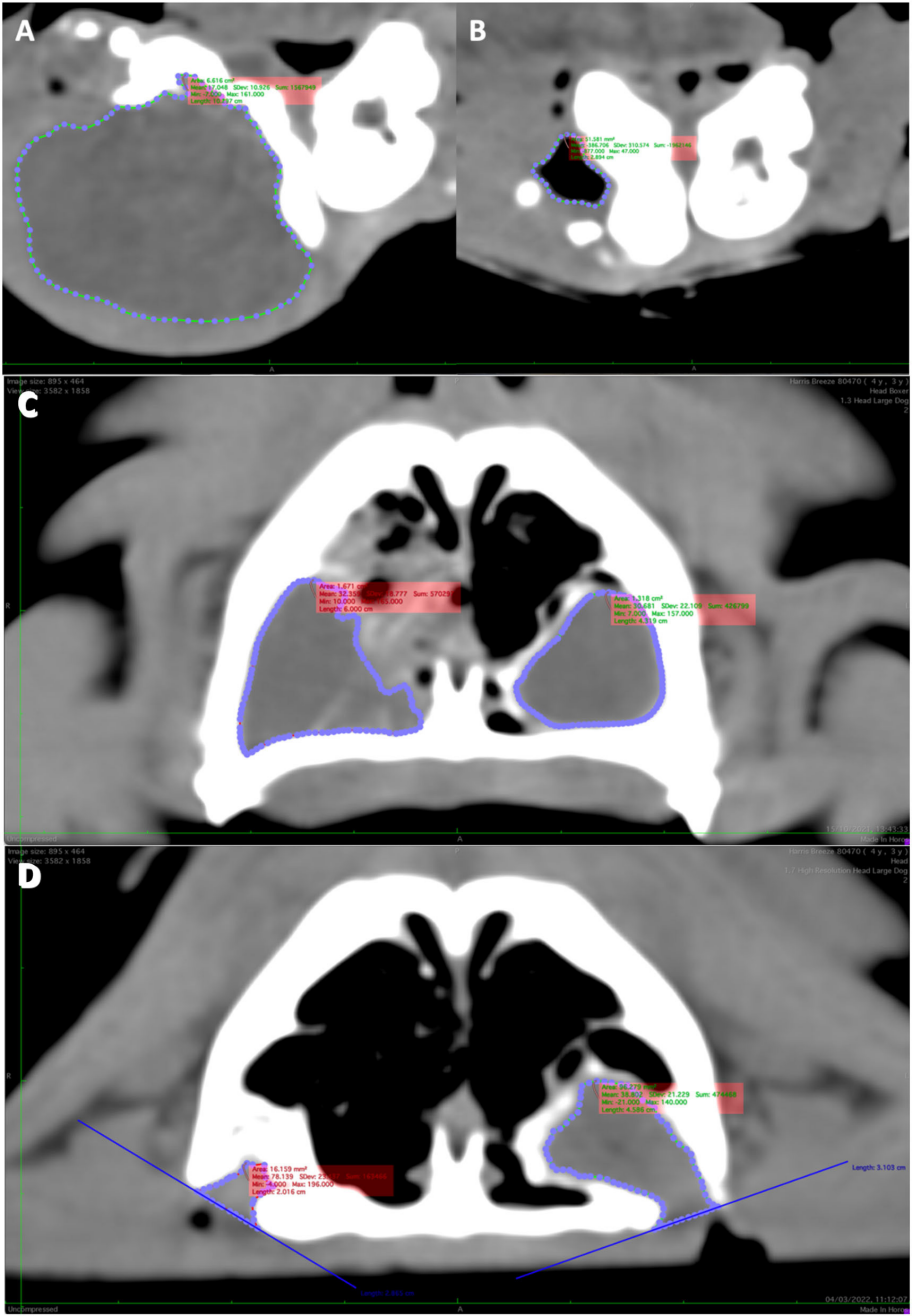
Reconstructed transverse CT scan demonstrating Region of interest (ROI) of a large right mandibular dentigerous cyst in Case 1 at time of presentation **(A)** and 92 days post-operatively showing a large reduction in cyst volume **(B)**. **(C)** The ROIs of two large cysts in the left and right maxilla of Case 5 and 141 days post-operatively **(D)**. Note the difference reduction in volume between the left and right cysts. **(D)** Also shows how the ROI was created in the location of the marsupialisation by drawing a straight line between the bone edges and using this as a guide.

To calculate the volume of each cyst, every CT scan was viewed in a transverse plane using the soft tissue window. The soft tissue windowing allowed clear delineation between the contents of the cyst and surrounding structures. The outline of each cyst was marked by manual placement of a series of points at the interface between cystic contents and the surrounding tissues using the closed polygon tool. These points were automatically linked together by the viewing software, creating a Region of Interest (ROI) which represented the outline of the cyst in that selected slice (Figure 2). This was then repeated for each slice within which the cyst appeared. In cases where the marsupialisation site was within the slice, the cyst was no longer enclosed, meaning that the entire cyst cavity could not be outlined. To solve this, a straight line was created between the outer margins of the bone at the site of the stoma. This line was used to represent the cyst lining at this point and incorporated into the ROI outline (Figure 2D). From this series of ROI polygons, an 3D representation of the cyst was created. The volume (cm^3^) was then calculated with the ROI compute volume function in the viewing software which uses a combination of the slice width, slice spacing and individual ROI volumes. The volume for each cyst was recorded at time of marsupialisation and at follow-up. The reduction in volume (cm^3^) between marsupialisation and follow-up was calculated for each cyst (Table 1). Each ROI polygon was created by the same individual and both the resident and radiologist were not blinded to the study.

**Table 1:** Summary of results.

**Cases**	**Age at presentation**	**Cyst location**	**Cyst category**	**Histological diagnosis**	**Cyst volume pre-treatment (cm^3^)**	**Cyst volume post-treatment (cm^3^)**	**Period between initial surgery and follow-up (days)**	**Reduction in cyst volume**
1	9 y 7 m	Right mandible	Bone + ST	Dentigerous	19.49	1.44	92	92.6%
2	2 y 4 m	Right mandible	Bone	Dentigerous	4.99	1.29	134	74.2%
		Left mandible	Bone	Dentigerous	2.19	0.08	134	96.2%
		Left mandible	Bone + ST	Dentigerous	7.12	0.41	134	94.2%
3	4 y 11 m	Left mandible	Bone + ST	Dentigerous	10.63	0.55	128	94.8%
		Right mandible	Bone	Dentigerous	4.64	2.49	128	46.4%
4	7 y 10 m	Right mandible	Bone	Dentigerous	0.58	0.49	160	13.9%
		Right mandible	Intranasal	Dentigerous	1.62	0.77	160	52.6%
5	3 y 6 m	Right mandible	Intranasal	Odontogenic	5.76	2.70	141	53.1%
		Right mandible	Intranasal	Odontogenic	7.69	0.31	141	96.0%
6	9 y 8 m	Right mandible	Bone	Dentigerous	5.29	4.46	144	15.7%
		Right maxilla	Bone + ST	Furcation	3.49	0.93	144	73.5%

Bone + ST, Bone with soft tissue involvement.

### Statistical analysis

Statistical analysis focused on the analysis of percentage reduction in cyst volume. Because several dogs had more than one cyst, generalized linear mixed effects models were used with dog as a random effect. The first steps of analysis were to consider whether the mean reduction differed from zero and to test separately the influence of each of five potential influencing factors (age, cyst location, cyst characteristics, cyst type, days post-marsupialisation). Any of these factors that achieved *p* < 0.20 were combined in an overall model, with nonsignificant terms then removed by backwards elimination. Residuals from this model met normality assumptions (Ryan Joiner test RJ = 0.977, *p* > 0.10). Residuals from this model met normality assumptions (Ryan Joiner test RJ = 0.977, *p* > 0.10). Tukey *post-hoc* tests were used to compare any significant factors with more than two levels. Significance was taken as *p* < 0.05, all analysis was undertaken in Minitab 19.

## Results

This case series consisted of six male Boxer dogs with a mean age of 6 years 3 months (range 2 years 4 months to 9 years 8 months). A total of 12 cysts were marsupialised. Fifty percentage (6/12) of the cysts were incidental findings with no evident clinical signs, with others presenting as facial swelling (3/12, 25%) and upper respiratory signs such as stertor and nasal discharge (3/12, 25%). The reason for pursuing marsupialisation was the presence of a cyst associated with teeth of strategical importance such as the canine, maxillary 4th premolar and mandibular 1st molar teeth (9/12, 75%). These teeth had no evidence of external inflammatory resorption, excellent periodontal health and the apical 1/3 of the root was encased in bone. The other three cysts were located intranasally where a complete enucleation would have required an advanced maxillofacial surgical approach such as a rhinotomy.

Seven cysts (58.3%) were in the mandible and five cysts (41.7%) were in the maxilla. Evaluation of the histopathology results and clinical findings provided a diagnosis of dentigerous cyst in 9/12 (75%). The remaining 3/12 (25%), were classified as odontogenic cysts with further classification not possible. An unerupted 1st premolar tooth was involved in 9/9 dentigerous cysts with 7/9 (77.8%) located in the mandible. Five of the cysts (41.7%) were completely surrounded by bone (B), 4 (33.3%) had extension into the surrounding soft tissue (B + ST), and 3 (25.0%) extended into the nasal cavity (B + NC). The mean volume of the cysts prior to marsupialisation was 6.13 cm^3^ (0.45–19.50cm^3^) and 1.32 cm^3^ (0.00–4.46cm^3^) at follow-up (Table 1). The mean reduction in cyst volume was 66.6% (13.9–96.2%) with a median of 74.2%. The mean time to initial follow-up CT scan was 138 days (median 141 days, range 92–160 days).

The reduction in cyst volume between pre-operative CT and follow-up CT was significantly different from zero (Table 2). An example of this volume reduction can be seen in Figure 2. Dog age, cyst category and days post-marsupialisation each had coefficients *p* < 0.20 (Table 2) and were considered in the final model. After backwards elimination this model consisted of age (with a coefficient of −0.017 %/day *p* = 0.018) and cyst category (estimated means for volume reduction adjusted for age: B 47.5%, B + ST 94.4% and B + NC 62.6%, *p* = 0.026). The only significant difference identified by a Tukey test was that the estimated volume reduction adjusted for age was significantly higher for cysts with extension into the surrounding soft tissue (B + ST) than cysts that were completely surrounded by bone (B).

**Table 2:** Initial analysis of overall reduction in volume and the influence of five potential factors on volume reduction.

	**Coefficient**	**Test statistic**	***p***
Overall mean reduction cf. zero	66.6%	*t*_5.05_ = 6.71	< 0.001
Age	−0.013 %/day	*F*_1, 10_ = 2.81	0.125
Cyst location	Mandible 58.7% Maxilla 78.1%	*F*_1, 8.2_ = 0.99	0.349
Cyst characteristics	ST and bone 85.4% Bone 46.3% Intranasal 70.9%	*F*_2, 7.6_ = 3.44	0.086
Cyst type	Dentigerous 70.3% Odontogenic 49.6% Furcation 89.5%	*F*_2, 5.3_ = 0.61	0.578
Period between initial surgery and follow-up (days)	−0.945 %/day	*F*_1, 10_ = 4.22	0.067

The size of the initial stoma ranged from 7 to 25 mm in diameter. All cases showed reduction in the size of the stoma site and there was no evidence of food impaction or infection of the stomas. There was no premature closure of any of the marsupialisation sites. At follow-up, all the cysts were enucleated.

## Discussion

Dentigerous cysts are the most common odontogenic cyst in dogs, occurring in 71% of cases (1). In a recent study by Bellei, brachycephalic dogs were over-represented in cases of unerupted teeth and 86% of unerupted teeth in Boxer dogs progressed to the formation of dentigerous cysts (7). The case series was limited to Boxer dogs, thus eliminating any breed specific variability which enabled us to determine the effectiveness of the treatment in Boxers.

Marsupialisation of an odontogenic cyst is a less invasive technique than extirpation. It allows immediate decompression of the cyst without excessive surgical trauma and trauma to surrounding structures. It also facilitates bone regrowth and remodeling which in turn causes shrinkage of the cyst (9). Marsupialisation can aid in reducing the size of large cysts where there is significant cortical bone destruction and concern about the risk of pathological jaw fracture (5, 11). This shows that the marsupialization technique is an effective method of reducing the volume of the odontogenic cysts examined. In smaller cysts, it can be useful when there is inclusion of a tooth of strategic importance and the approach to remove the cyst lining entirely would damage the tooth and surrounding structures.

Studies in humans have shown that after 100 days the volume of the cyst decreases at a slower rate (12). Given this information, a minimum follow-up period of 3 months was recommended for every marsupialisation case treated in this series.

Using computed tomography, the marsupialisation technique has been shown to be an effective preliminary treatment of odontogenic cysts, reducing the luminal volume of cysts by a mean of 66.6%. This reduction in volume was significant and supports findings of previous human studies (13–18). Kubota et al. showed that the pressure from intracystic fluid of Keratocystic Odontogenic Tumors in humans, stimulated release of inflammatory cytokines such as Il-1a from the epithelial cells which stimulated osteoclastogenesis and activated resorption of bone around the lesion (15). Although the process of cyst reduction is not fully known, this finding suggests that decompression of an odontogenic cyst prevents any further stimulation of the epithelial cells, and therefore any further release of inflammatory cytokines, allowing bone regrowth into the defect.

The rate of reduction of the cysts when adjusted for age was found to be significantly higher in cysts with extension into surrounding soft tissue (B + ST), compared to cysts that were completely surrounded by bone (B) and those which extended into the nasal cavity (B + NC). B + ST cysts were the largest in the case series with a mean volume of 10.8 cm^3^ (3.5–19.5 cm^3^). In comparison, B cysts had a mean volume of 3.5 cm^3^ (0.6–5.3 cm^3^) and B + NC cysts had a mean volume of 5.0 cm^3^ (1.6–7.7 cm^3^). This finding supports that of previous human studies which found that the larger the cyst, the greater the rate of volume reduction post-marsupialisation (14, 15).

Follow-up CT and radiographs at least indicated new alveolar bone formation around the teeth left in place and that the teeth were vital, however, the presence of periodontal ligament regeneration could not be confirmed. Histopathology would be required to determine whether there is periodontal ligament regeneration as a result of decompression and bone regrowth. It is uncertain whether that these teeth retain their periodontal ligament or whether it is destroyed as a result of cystic expansion.

Seventy-five percentage of the identified odontogenic cysts were dentigerous and were a result of an unerupted 1st premolar tooth. Given that brachycephalic dogs are predisposed to unerupted teeth and 86% of unerupted teeth in Boxer dogs progress to the formation of dentigerous cysts (7), a detailed oral examination and dental radiography of Boxer dogs with permanent dentition may be beneficial at a young age to identify unerupted teeth. These teeth could then be surgically extracted before cyst formation.

Oral home care and frequent follow-up examination were very important in the post-operative management of the stoma. Therefore, good owner and dog compliance is a key component in the decision process when considering performing a marsupialisation. Prior to treatment, all clients were given information on the marsupialisation technique, aftercare and the follow-up required. Appropriate oral home care was then demonstrated at discharge. The post-operative protocol used for each of the cases in this study was a 2 week duration of twice-daily lavage of the marsupialisation sites with 0.12% chlorhexidine solution, which was based on previous reports in humans (6, 9, 19, 20). Other solutions have been used post-operatively including distilled water (21) and normal saline (9, 22). In human dentistry, the cyst cavity is packed with gauze for 2–4 weeks in addition to post-operative lavage. The gauze is replaced on a weekly or biweekly basis and is soaked in a topical antibacterial solution such as Iodoform (21, 23, 24), Fusidic acid (9), or Bacitracin (22). We chose not to pack the stoma of the cyst with gauze in these cases, as they would need regular replacement, and this may require multiple sedations or general anesthetics depending on the temperament of the dog.

In human patients, marsupialisation sites are reported to be uncomfortable, especially in the early stages (25). For this reason, a multimodal analgesia protocol was used postoperatively in these cases to prevent any discomfort.

Regular follow-up was advised to monitor the stoma site and the effectiveness of the oral home care performed by the owner. If premature closure did occur, reopening of the stoma under general anesthetic would be required. Communication was crucial and clear instructions on follow-up examination and treatment were supplied to the referring vet to avoid confusion and complications during initial follow-up. Causes of premature closure may include the size and location of the stoma, as well as the characteristics of the surrounding tissues incorporated into the stoma.

Neoplastic transformation of cyst lining to an ameloblastoma and a squamous cell carcinoma have previously been reported in odontogenic cysts. Histopathological examination of the cyst lining was performed to confirm the diagnosis of a cyst and identification of any neoplastic transformation of the cyst lining (11, 27). However, only a small sample of cyst lining was obtained which may not be representative of the cyst and therefore there is a potential to miss a neoplastic lesion.

The open source imaging software (Horus, OsiriX^TM^, Geneva, Switzerland) was chosen to measure preoperatively and at follow-up for each of the cases in this series. The method used to determine the volume is known as manual segmentation. In a recent study, manual segmentation using Horos was compared against alternative techniques and software in the assessment of pre- and post-operative tumor volumes of high-grade gliomas in humans. It was found that the manual and semi-automated software showed high agreement in preoperative volumetric assessment and were adequate for post-operative volumetric assessment (26). From this conclusion, we were confident that the software tool could be used to measure the pre- and post-operative cystic volumes accurately.

A major difficulty encountered during this study was determining the region of interest at the location of the marsupialisation site. At this point the cyst lining is continuous with the oral cavity and not self-contained. For this study, it was agreed that the region of interest should extend to the level of the stoma. The level of the stoma was determined by creating a straight line across the point where the cyst lumen is exposed to the oral cavity between the two outermost points on the surrounding bone. This method was used for each slice in which the marsupialisation site was present for every follow-up CT scan.

The main limitation of this case series is that it is a retrospective case series and therefore factors such as period between initial surgery and follow-up CT scan could not be controlled. We found that using contrast computed tomography allowed clearer delineation between the cyst and the surrounding structures. However, only two of CT scans in this case series made use of contrast enhancement. Therefore, all of the cysts were analyzed and measured using the soft tissue window without contrast enhancement to reduce variability and give a more consistent result. Contrast enhancement may have allowed more precise measurement of the cyst volume. However, in the authors opinion, the use of contrast may not have contributed to the overall result given the significance of the volume reduction determined from the current data.

Since only Boxer dogs were included in the study, there may be variations in the response to marsupialisation between different breeds, meaning that the results of this study should be applied with caution to the treatment of dogs of other breeds. Another limitation of this study was the small number of cases included in this case series. A larger study would allow further cross comparison between the location of the cysts and the volume reduction post-marsupialisation as well as a direct comparison between types of cysts. The effect of age, sex and breed could also be investigated in detail in a larger sample.

## Conclusion

This case series shows that marsupialisation is an effective, non-invasive 1st stage treatment of odontogenic cysts. The effect of decompression through the use of marsupialisation of the cysts causes a reduction in the volume of the cysts. Extirpation can be performed as a second stage treatment, once the cyst is smaller, with reduced risk of iatrogenic damage to vital structures.

## Data availability statement

The original contributions presented in the study are included in the article/supplementary material, further inquiries can be directed to the corresponding author.

## Ethics statement

Ethical review and approval was not required for the animal study because retrospective case series on a surgical technique already used routinely by the authors. Written informed consent for participation was not obtained from the owners because this is a retrospective case series where the procedure had been performed prior to the study's conception.

## Author contributions

JH wrote the manuscript, conceived and designed the study, performed the manual segmentation on all CT scans, and contributed to the statistical analysis. IT and PS contributed to conception and design of the study. All authors read the manuscript, contributed to manuscript revision, and approved the submitted version.

## References

[1] Verstraete FJM Zin BP Kass PH Cox DP Jordan RC. Clinical signs and histologic findings in dogs with odontogenic cysts: 41 cases (1995-2010). J Am Vet Med Assoc. (2011) 239:1470–6. 10.2460/javma.239.11.1470 22087723

[2] Murphy BG Bell CM Soukup JW. Veterinary Oral and Maxillofacial Pathology. Wiley-Blackwell (2019). 10.1002/9781119221296

[3] Wakolbinger R Beck-Mannagetta J. Long-term results after treatment of extensive odontogenic cysts of the jaws: a review. Clin Oral Investig. (2016) 20:15–22. 10.1007/s00784-015-1552-y 26250795

[4] Anavi Y Gal G Miron H Calderon S Allon DM. Decompression of odontogenic cystic lesions: clinical long-term study of 73 cases. Oral Surg Oral Med Oral Pathol Oral Radiol Endod. (2011) 112:164–9. 10.1016/j.tripleo.2010.09.069 21194990

[5] Doran I Pearson G Barr F Hotston-Moore A. Extensive bilateral odontogenic cysts in the mandible of a dog. Vet Pathol. (2008) 45:58–60. 10.1354/vp.45-1-58 18192577

[6] Torres-Lagares D Segura-Egea JJ Rodríguez-Caballero A Llamas-Carreras JM Gutiérrez-Pérez JL. Treatment of a large maxillary cyst with marsupialization, decompression, surgical endodontic therapy enucleation. J Can Dent Assoc. (2011) 77:6–11. 21736863

[7] Bellei E Ferro S Zini E Gracis M. A clinical, radiographic and histological study of unerupted teeth in dogs and cats: 73 cases (2001–2018). Front Vet Sci. (2019) 6:357. 10.3389/fvets.2019.00357 31788479 PMC6856145

[8] Partsch C. Uber kiefercysten. Dtsch Monatsschrift Fur Zahnheilkd. (1892) 10:271.

[9] Abu-Mostafa N Abbasi A. Marsupialization of a large dentigerous cyst in the mandible with orthodontic extrusion of three impacted teeth. A case report. J Clin Exp Dent. (2017) 9:e1162–6. 10.4317/jced.53890 29075422 PMC5650222

[10] Kortegaard HE Reiter AM Legendre L Eriksen T Buelund LE Gorrel C. Marsupialization followed by curettage of an extensive periapical cyst in the incisive and maxillary bone in a dog. J Vet Dent. (2018) 35:268–74. 10.1177/0898756418813645

[11] Soukup JW Lawrence JA Pinkerton ME Schwarz T. Computed tomography-assisted management of a mandibular dentigerous cyst in a dog with a nasal carcinoma. J Am Vet Med Assoc. (2009) 235:710–4. 10.2460/javma.235.6.710 19751168

[12] Miyawaki S Hyomoto M Tsubouchi J Kirita T Sugimura M. Eruption speed and rate of angulation change of a cyst-associated mandibular second premolar after marsupialization of a dentigerous cyst. Am J Orthod Dentofacial Orthop. (1999) 116:578–84. 10.1016/S0889-5406(99)70192-7 10547520

[13] Nakamura N Mitsuyasu T Mitsuyasu Y Taketomi T Higuchi Y Ohishi M. Marsupialization for odontogenic keratocysts: long-term follow-up analysis of the effects and changes in growth characteristics. Oral Surg Oral Med Oral Pathol Oral Radiol Endod. (2002) 94:543–53. 10.1067/moe.2002.128022 12424446

[14] Consolo U Bellini P Melini GM Ferri A Lizio G. Analysis of marsupialization of mandibular cysts in improving the healing of related bone defects. J Oral Maxillofac Surg. (2020) 78:1355.e1–11. 10.1016/j.joms.2020.02.034 32482564

[15] Kubota Y Imajo I Itonaga R Takenoshita Y. Effects of the patient's age and the size of the primary lesion on the speed of shrinkage after marsupialisation of keratocystic odontogenic tumours, dentigerous cysts, and radicular cysts. Br J Oral Maxillofac Surg. (2013) 51:358–62. 10.1016/j.bjoms.2012.07.017 22981336

[16] Marker P Brøndum N Clausen PP Bastian HL. Treatment of large odontogenic keratocysts by decompression and later cystectomy: a long-term follow-up and a histologic study of 23 cases. Oral Surg Oral Med Oral Pathol Oral Radiol Endod. (1996) 82:122–31. 10.1016/S1079-2104(96)80214-9 8863300

[17] Zhao Y Liu B Han QB Wang SP Wang YN. Changes in bone density and cyst volume after marsupialization of mandibular odontogenic keratocysts (keratocystic odontogenic tumors). J Oral Maxillofac Surg. (2011) 69:1361–6. 10.1016/j.joms.2010.05.067 21195525

[18] Bodner L Bar-Ziv J. Characteristics of bone formation following marsupialization of jaw cysts. Dentomaxillofac Radiol. (1998) 27:166–71. 10.1038/sj.dmfr.4600344 9693529

[19] Maltoni I Maltoni M Santucci G Ramina F Lombardo L Siciliani G. Marsupialization of a dentigerous cyst followed by orthodontic traction of two retained teeth: a case report. Int Orthod. (2019) 17:365–74. 10.1016/j.ortho.2019.03.019 31023587

[20] De Azambuja Berti S Pompermayer AB Souza PHC Tanaka OM Westphalen VPD Westphalen FH. Spontaneous eruption of a canine after marsupialization of an infected dentigerous cyst. Am J Orthod Dentofac Orthop. (2010) 137:690–3. 10.1016/j.ajodo.2009.10.023 20451790

[21] Hassan KS Marei HF Alagl AS. Composite bone graft for treatment of osseous defects after surgical removal of impacted third and second molars: case report and review of the literature. Oral Surgery, Oral Med Oral Pathol Oral Radiol Endodontology. (2011) 112:e8. 10.1016/j.tripleo.2011.04.010 21784674

[22] Anthony Pogrel M Jordan RCK. Marsupialization as a definitive treatment for the odontogenic keratocyst. J Oral Maxillofac Surg. (2004) 62:651–5. 10.1016/j.joms.2003.08.029 15170272

[23] Ertas U Yavuz MS. Interesting eruption of 4 teeth associated with a large dentigerous cyst in mandible by only marsupialization. J Oral Maxillofac Surg. (2003) 61:728–30. 10.1053/joms.2003.50145 12796888

[24] Qian WT Ma ZG Xie QY Cai XY Zhang Y Yang C. Marsupialization facilitates eruption of dentigerous cyst-associated mandibular premolars in preadolescent patients. J Oral Maxillofac Surg. (2013) 71:1825–32. 10.1016/j.joms.2013.06.223 23973048

[25] Briki S Elleuch W Karray F Abdelmoula M Tanoubi I. Cysts and tumors of the jaws treated by marsupialization: a description of 4 clinical cases. J Clin Exp Dent. (2019) 11:e565–9. 10.4317/jced.55563 31346379 PMC6645260

[26] Zeppa P Neitzert L Mammi M Monticelli M Altieri R Castaldo M. How reliable are volumetric techniques for highgrade gliomas? A comparison study of different available tools. Neurosurgery. (2020) 87:E672–9. 10.1093/neuros/nyaa282 32629469

[27] Angelopoulos AP Tilson HB Stewart FW Jaques WE. Malignant transformation of the epithelial lining of the odontogenic cysts: Report of a case. Oral Surg Oral Med Oral Pathol. (1966) 22:415–28. 10.1016/0030-4220(66)90420-8 5222625

